# Exploring residents’ experiences of mealtimes in care homes: A qualitative interview study

**DOI:** 10.1186/s12877-017-0540-2

**Published:** 2017-07-11

**Authors:** Ross Watkins, Victoria A. Goodwin, Rebecca A. Abbott, Abi Hall, Mark Tarrant

**Affiliations:** 1 University of Exeter Medical School, St Luke’s Campus, Heavitree Road, Exeter, EX1 2LU UK; 2 National Institute for Health Research (NIHR) Collaboration for Leadership in Applied Health Research and Care (CLAHRC), South West Peninsula, UK

**Keywords:** Residential care, Older adults, Mealtimes, Semi-structured interviews

## Abstract

**Background:**

Many interventions aim to alleviate well-documented problems of malnutrition in residential care homes and improve residents’ health and wellbeing. Despite some positive findings, little is known about *how* and *why* mealtime interventions might be effective, and in particular, what effects residents’ experiences of mealtimes have on health outcomes. The aim of this study was to gain an insight into these experiences and explore some of the issues that may impact on residents’ enjoyment of meals, and resulting health and wellbeing.

**Methods:**

Semi-structured interviews were conducted with eleven residents from four care homes in the South West UK. Thematic analysis was used to derive content and meaning from transcribed interviews. Interviews were supplemented by researcher observations of mealtimes.

**Results:**

The dining experience was a focal point for participants’ broader experiences of residing in a care home. Three themes pertaining to residents’ experiences were identified: (1) Emotional and psychological connections with other residents; (2) managing competing interests with limited resources; and (3) familiarity and routine.

**Conclusion:**

Mealtimes are a mainstay of life in a care home through which residents’ experiences are characterised, exemplified and magnified. Understanding how residents interact with one another, accommodating their preferences and encouraging autonomy may enhance their mealtime experiences. It may also help to ease the transition from independent-living to life in care, which can be particularly stressful for some residents, and improve health and wellbeing over the long-term.

## Background

More than 400,000 older adults in the UK live in care [1], an estimated 60% of whom are aged 85 or over [2]. Residential provision for these adults is generally referred to as a care home, defined as a setting in which residents usually have a single room and access to on-site care services, and including those care homes with nursing services [3]. Regardless of their specific classification, there is considerable overlap in the health status and clinical needs of this population across settings. In England, approximately 75% of care homes are privately-owned, 15% are owned by the voluntary sector, and 10% are public sector [4]. Around 70% of the care home market is state-funded [4]. The health and wellbeing of care home residents is of ongoing concern. Effects of underlying medical conditions in older people are compounded by low mood, depression, anxiety, and loneliness [5], contributing to an often poor quality of life among care home residents [6]. In the UK, the incidence of depression in care homes is particularly high, estimated to affect almost one third of residents, three times the proportion estimated to be affected in the community-dwelling population [7]. A common side-effect of poor psychological or emotional health is a dwindling appetite and a decline in nutritional status [8]. For instance, depression and apathy have been independently associated with weight loss in care home residents [9].

The current study investigated care home residents’ experiences of their care, with a particular focus on their experiences of mealtimes. Mealtimes are an integral part of day-to-day life in a care home [10] and are a pivotal point for the delivery of care. The mealtime experience may therefore be an important catalyst for the health, wellbeing and quality of life of residents. Yet, a recent systematic review concluded that there is a paucity of research pertaining to the resident experience of mealtimes in care homes [11]. Building on existing research that suggests a positive effect of mealtime interventions on nutritional outcomes of residents and the behaviour symptoms of people with dementia [12, 13], this study sought to address this gap by investigating the experiential component of mealtimes. This reflects Medical Research Council (MRC) guidance on developing and evaluating complex interventions which highlights the importance of establishing a theoretical understanding of how interventions work [14].

Mealtimes represent more than just the provision of nutrition; they may offer residents (and staff) the opportunity to form and sustain important social relationships. Food is used to provide comfort, express feelings, celebrate or reward success, and nurture companionship [15]. Eating occasions are integral to tradition, to family life, and to identity [16]. In stressful situations or in unfamiliar environments, or indeed when the notion of identity is compromised, food (and the social connections to it) may significantly influence quality of life [17]. Whilst it is acknowledged that mealtimes have a critical socio-cultural role in the care of older people, existing interventions are characterised by their focus on single components, and lack the complexity associated with health and wellbeing determinants [12, 13, 18]. For example, a nutrition-based intervention such as the provision of snacks between meals may not be effective in the long-term if interest in eating is poor or residents are not skilfully assisted. Similarly, an intervention based on altering the design of the dining room or changing the way in which food is served, does not ensure that the dining experience will be pleasant or that the social aspect of eating will be enhanced. Prior research has indicated that residents can feel disenfranchised in their care home, manifested in a perceived loss of control [19], as routine decisions are taken away from them and staff adopt paternalistic approaches to care provision at mealtimes [20]. This negates a key element of person-centred health care and social care as defined by the Care Quality Commission (CQC), which advocates giving people choice and control over their own care, treatment and support [21].

Enabling resident choice or personal preferences is difficult in care homes because many residents choose not to draw attention to their negative experiences of care. In a study conducted in ten Australian nursing homes, Pearson et al. [22] observed that residents reported not wanting to be labelled as “whiners” and not wanting to inconvenience staff. Reimer et al. [20] describe care home residents as “silent recipients of care” as they tend not to raise concerns or express preferences about mealtimes, either because severe cognitive decline leaves them unable to do so, or because it is engrained within the cultural values of their generation [23]. The absence of verbalised dissatisfaction cannot necessarily be taken as an indicator of satisfaction and warrants further investigation into care home residents’ experiences of their care. Such an investigation may help identify and develop a basis for future interventions in care homes [14]. The current study aimed to:

1. Gain an insight into residents’ perspectives on mealtimes in care homes;
2. Elicit some of the important issues that impact on residents’ dining experiences, including how their social interactions may affect their enjoyment of meals.

## Methods

Ethical approval for the study was given by the authors’ Research Ethics Committee (Reference Number: 15/07/075). Written consent was obtained from all participants prior to interviews.

### Sampling of care homes

In England, care homes for adults are regulated by the Care Quality Commission (CQC), which carries out regular inspections to ensure that care is safe, effective and compassionate, and that improvements are made where necessary [3]. Care homes rated by the Care Quality Commission (CQC) as inadequate or requiring improvement were not selected for inclusion in the study. A purposive sampling approach was used to select the participating care homes. This type of sampling is not intended to generalise to the population as a whole, but rather identify common links or characteristics between the observed setting and other settings like it [24], and reflect the diversity within the care home population [25]. This is a standard approach to sampling in qualitative research. In the current study, the criteria for a typical case was to include privately-run, mid-size care homes from both a rural and urban locale. Recruitment took place through existing research networks at the lead researcher’s institution, including PENCLAHRC’s network of contacts for patient and public involvement in research (PPI). Care Home Managers in selected homes were sent a letter inviting them to take part in the study. The letter provided managers with some details about the study. The lead researcher then made an initial visit to interested care homes to discuss the study in more detail. Care home staff were given copies of the participant information sheet, which they could discuss with prospective participants. Once potential participants had been identified by care home staff, and any queries or questions about the study had been addressed, the lead researcher liaised with the Care home Manager about a suitable date and time to conduct the interviews. A key objective in the recruitment process was to ensure that the research did not detract from the provision of planned care in the sampled homes.

### Participants

Male and female residents aged 65 years or older from selected care homes were invited to take part in this study. Whilst the care homes in this study also accommodate residents with mild cognitive impairment (MCI) or a diagnosed form of dementia, care home staff assisted in the recruitment of participants who were likely to be suitable candidates for interview and able to give independent and informed consent. As their primary care-givers, care home staff were best placed to assess whether residents were cognitively able to give independent consent. These were residents who were able to articulate their experience of mealtimes, as this was integral to the research. Informed by previous studies, it was expected that between ten and fifteen participants would be needed in order to give a sufficient range of experiences and depth of data to reach theoretical saturation [26, 27], the point at which no new data emerges to provide additional insights into the research question [28]. Each participant who gave their consent to take part in the study was assigned a unique reference number (e.g. RES01).

### Semi-structured interviews

Interviews were conducted by the lead researcher (RW) and focused on the experience of mealtimes, including the social environment in which they take place. The interview strategy was designed to facilitate a coherent discussion, with participants free to say as much or as little as they wished. Each interview was conducted face-to-face in a private setting in the participating care home, and lasted approximately 20–30 min. In order to provide context to the participants and the researcher, interviews took place in the dining room between meals where possible. Only the lead researcher and resident participant were present during each interview.

As new issues or themes emerged in the interviews, they were included in subsequent interviews and structured further questioning. This approach was inspired by Grounded Theory [29] in which a theory emerges iteratively and develops through the analysis of data. As data is collected, repeated ideas (e.g., views and opinions) are tagged with codes, which can then be grouped into concepts and/or categories. Interviews were audio recorded and transcribed. During each interview, nonverbal expressions and gestures were recorded in the interviewer’s field notes in order to enable a more detailed description of the conversation and give further insight into a participant’s perspective. The field notes contained the researcher’s observations and thoughts about the atmosphere and interaction, contributing to a “thick description” of the data [30]. These field notes were also used during data analysis to note thoughts and emerging insights.

### Observations and field notes

Prior to conducting interviews, mealtimes were observed at each of the care homes. This was non-participatory and served to provide context to the participants’ interview data. Care home managers provided verbal consent for these observations. Field notes from these observations were anonymised for use in subsequent analysis and reporting.

### Data analysis

Interview data were analysed using Thematic Analysis [31, 32]. The aim of the analysis was to organise the data in a meaningful way so as to develop theory about the forms, functions and consequences of mealtime experiences in the care home environment.

The organisation and analysis of data followed the steps outlined below:

1. *Familiarisation with the data* – Listening and reading through the data. The first coder was also the interviewer.
2. *Generation of initial codes* – Naming key features of the data.
3. *Searching for themes* – Grouping codes into potential themes.
4. *Reviewing themes* – Ensuring that the themes are distinct from other themes and internally coherent and consistent.
5. *Defining and naming themes* – Interpreting and giving the themes analytically meaningful names. Extracts that represent the essence of the respective themes were identified in this step.
6. *Generating a thematic network* – Mapping interconnection between the themes
7. *Producing Theory and Report* – Interpretation and reporting the themes and the interconnections between them beyond description and ensuring that all analytical claims are congruent with the extracts.

Two researchers (RW, AH) familiarised themselves with the whole data set and following initial familiarisation with the transcripts, developed a bank of codes. The researchers then coded the transcripts independently and compared analyses, with any differences resolved through discussion. Following this, the lead researcher (RW) organised the coded data into themes, which were reviewed by a second reviewer (AH). Differences were resolved through discussion of the themes. NVivo 10 (QSR International) was used to help organise and code the data. The provisional themes were then refined after discussion with all of the authors. To ensure that potential biases did not occur on the part of the lead researcher, a research diary was also kept. This enabled a reflexive approach to data collection and analysis [33], and provided insight which in turn help to inform data analysis. This is a well-established technique for improving the quality of the emerging explanations [34].

## Results

**Fig. 1:**
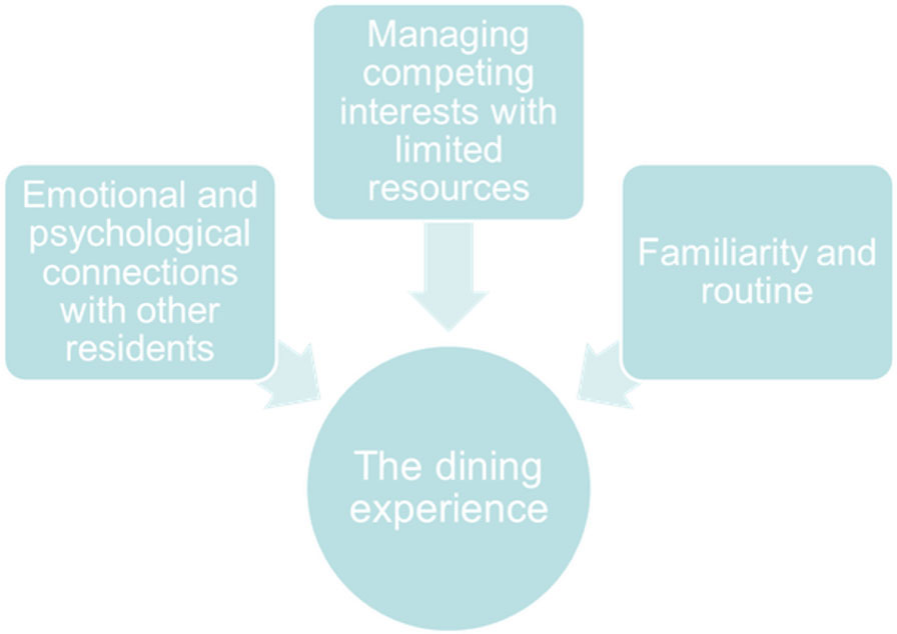
Experiential components of the mealtime

Six women and five men were recruited from four care homes. Further recruitment was not undertaken as new themes in the data were not emerging. The age of participants ranged from 78 to 97 with a mean age of 87. None of the participants required feeding assistance. Two participants described having hearing difficulties, which effected conversation with their table companions. One participant was diabetic, which restricted food choice. The care homes were all privately-run, small to medium in size with a bedroom number ranging from 18 to 46. As well as describing various organizational and procedural aspects of them, participants gave nuanced accounts of their dining experiences which they linked to their broader experiences of life in care. Three themes emerged from the analysis pertaining to these experiences: [1] Emotional and psychological connections with other residents; [2] managing competing interests with limited resources; and [3] familiarity and routine (see Fig. 1).

Although these themes reflect residents’ experiences of care in general, it was clear that mealtimes are a focal point for these experiences. Anonymised participant quotes are used to illustrate themes. Interviewer field notes are used to elaborate on the context and meaning of themes derived from participant responses.

### Emotional and psychological connections with other residents

Participants discussed their social interactions in the care home, the nature of their relationships with other residents, and the implications that living in a care home community has for these relationships. Mealtimes were viewed as an opportunity to establish and maintain relationships with other residents, but it was acknowledged that communication difficulties provided a barrier to this. Relationships were also highly influenced by tablemate interactions. Therefore, staff had an important role in facilitating emotional and psychological connections between residents. For some of the participants, the transition from independent living to living in a care home had been difficult, but they had adjusted to it by developing positive relationships with other residents:

*“You see, it might not work for everyone, but I don't know anyone here who's not happy here. You can tell from their faces. But the main reason for me is to find myself in a happy community. It's not as interesting as I might dream of, but so were a lot of jobs I had initially.”* (RES07)

In each of the four homes, the dining room represented a significant communal meeting place for residents, some of whom may not have seen each other throughout the rest of the day. Even in participants for whom communication was physically limited, there was a desire to build relationships and a sense of community: *“I wished I could hear better, the conversation is limited only because our lives are limited now”* (RES07). Although there was an implicit acceptance of these difficulties, there was a desire to improve communication, and in so doing forge a renewed sense of community:

*“I mean, it troubles me a little in that the three blokes who sit with me - or I sit with the three blokes - we're all in different stages of an illness which prevents communication. And I've been racking my brains as to how I might not sort of impose anything, but enquire of the girls, the care assistants, how we might go about changing it.”* (RES08)

Moreover, participants made it clear that social interaction is more than verbal communication, and that despite their communication difficulties, they had a “shared understanding” or an “unspoken bond” between them, and that irrespective of their background and stage of illness, they had their “humanity in common”. A sense of community, however it was manifested, enabled residents to feel emotionally and psychologically connected. However, the connected community could be interrupted on occasion by the abrasive personality of some residents or by the challenging behaviour that accompanies some types of dementia:

*“There’s (resident’s name), she sits over there and we can hear her moaning about the food all the time, and she’ll go at them, and one day, they put her out the room. She doesn’t help out, because she makes her life a misery and no-one can’t get on with her.”* (RES04)

Tablemate interactions were a key factor in establishing emotional and psychological connections, as residents reported that they sat in the same place for each meal surrounded by the same table companions. Staff had an important role to play in facilitating social relations, either by enabling appropriate table groupings or by sitting down and eating with residents to support conversation. The extent of social interaction was observed to be quite mixed between residents and amongst the different care homes. Environmental factors such as menus, table service, interaction with staff, and mealtime themes were often a trigger for conversation. For instance, in one home it was observed that dessert was chosen from a trolley, and this gave the residents an opportunity to ask questions about the dessert selection and discuss their options with their table companions.

### Managing competing interests with limited resources

Participants alluded to the importance of individual choice, including menu choice, and acknowledged the trade-off between catering for personal preferences and the constraints of the collective provision of meals. Whilst participants valued their autonomy, including what, when and with whom to eat, they recognised that there were competing interests between the personal preferences of residents and the ability of care home staff to accommodate these preferences, particularly given their limited resources. Moreover, the balance between allowing residents to be independent and ensuring the care home provided residents with the necessary support was challenging. Some participants felt they had enough autonomy: *“You don't have to do anything you don't want”* (RES11), whilst others acknowledged the importance of support, particularly around mealtimes, and stressed that independence is not always desirable:

*“I think what happens in these establishments is that if people are not able, they're left to their own devices to see if they can actually do it - to encourage independence. And there can be the danger, I think, of not eating as much as you should, and then saying enough is enough, because you simply haven't got the strength or otherwise to finish off the meal or cope with it yourself.”* (RES08)

There was a general acceptance that meal choice was necessarily limited by the collective provision of food and the diversity of personal preferences: *“They (care home staff) can't please everybody all the time, can they? I mean, they get to know what people like. As I say, I don’t like curries, but most of them do have a curry”* (RES10). This resulted in meal options being described as “hit and miss”*,* with participants conceding that it was difficult for collective provision to replicate the satisfaction derived from a home-cooked meal:

*“Yes, so you look at the menu and see “ooh its sausage and mash today, oh that’s alright”, and another day it might be something else, and you think, “oh, I’m less keen on that”, you know, but at home it’d be unusual to turn your nose up at anything.”* (RES06)

Some participants expressed a preference for traditional, culturally-familiar foods, and a dislike for those that were unfamiliar: *“I said to them, in a nice way, “Look, I don’t eat nothing else but English food and I’m not going to start it, I’m sorry””* (RES04). Thus, when encouraged to try new foods, individuals may sometimes resist the imposition of collective provision – a tension that can arise from competing interests. Providing residents with a choice at mealtimes helped to resolve the tension and avoid conflict:

*“We're given a menu, two things a day, but half of it's things I never eat. So I stick to the things I know … we had sausage and mash and beans today. Then we had something with jam on, I don't know what it was, it was quite nice. We have two things.”* (RES09)

The mealtime provided individual choice in one way, but in other ways was seen as restrictive. For instance, residents were typically assigned a seat at a table on admission to the care home based on availability and on the level of assistance they required. Invariably, this became *their* seat: *“We can sit wherever we like, in theory, but we tend to end up in the same place”* (RES02). Therefore, whilst this structure facilitated social interaction, the lack of choice over where to sit inhibited residents’ opportunity to establish their own relationships. Table allocation was also an important part of the induction process for new residents and a determinant of subsequent companionship. Although this illustrates how the transition into care may be eased for new residents, in similar scenarios individuals seated together may not necessarily bond with each other:

*“Yep, I’m always on this table. There was a lady just joined us, who was new and they asked me if I would keep her company, and said “Yeah, I can do that” because I talked to her daughter beforehand and she said, “I’ve been told by the head one here that you’re very good with newcomers, so will you look to my Mum”, and I said, “Yeah”. We get on ever so well together.”* (RES04)

There was a shared dissatisfaction over the delay in service, either before the meal or between courses, with participants also conveying frustration at the attitude of staff. This engendered an expression of collective disenchantment, although this had positive implications for residents’ social identity:

*“I think… the other day I was annoyed, um, the person on the table with me complained about waiting. And they said well go to the restaurant down the road and see if they keep you waiting so long. That was one of the girl’s cheeky reply, you know, but typically speaking I would expect better service in a restaurant. In that respect, the waiting business … So we sit there waiting and waiting and the clocks on the wall saying ten minutes, twenty minutes, 30 minutes, sometimes its three quarters of an hour, you know, you’re waiting.”* (RES06).

In addition, there was a collective sense amongst participants that delays in service were due to a shortage of staff at mealtimes, and that providing care to individual residents was limited by resource pressures: *“Well, they tell you that they're really understaffed, they really want more staff. But I suppose if they had more staff, the prices would go up even more”* (RES10). One participant remarked that staff had several responsibilities during mealtimes, including serving as waiting staff which could detract from their main responsibilities: *“(Catering Manager) said in an ideal world he would like a waiting staff, so the carers could do their caring and the waiters could do the waiting, because the carers say they’re not waiters or waitresses, which is fair enough”* (RES02). Despite this, participants appreciated the staff efforts to be attentive and on-hand to deal with their individual needs.

### Familiarity and routine

Participants inferred that habits and routine had a key influence on their experience of mealtimes, as well as their broader experience of life in a care home. Residents’ habitual behaviour had developed over a lifetime, and as a result was perhaps more entrenched. Whilst they may be less inclined to deviate from habitual norms, participants appreciated the opportunity to mark special occasions, especially if this offered them a chance to reminisce. Participants discussed how they spent a typical day in the care home, and alluded to some of their personality traits and how these impacted on their experience of life in residential care. For residents who preferred to keep their own company and who were less willing to participate in other group activities (e.g., bingo or quizzes), mealtimes broke up the day and provided an opportunity to build and maintain social relations with others.

Participants appeared to find comfort in familiarity and routine, and this was often reflected in their meal choice: *“Tends to be the same breakfast each day. I don't find anything wrong with that, I'm a creature of habit anyway”* (RES08). This exemplifies how routine can represent personal preferences. Many of these preferences are based on long-standing habits and well-established rituals:

*“At lunch by my choice I only have soup. I'll tell you why. Because all my life I've been on the run, grabbing a sandwich and so forth, so I'd never eat at lunch at all. I mean, they have a very lovely lunch, but I only have soup.”* (RES07).

The ritualistic aspect of mealtimes was recognised by staff in all of the homes, which regularly focussed efforts on the marking of special occasions. Resident and staff birthdays were celebrated with a cake, and in one home, residents could mark their birthday by deciding what food options were offered on the lunch menu: “*… on birthdays you’re given a cake and the menu is your choice that day. Then, they bring in the cake and it’s cut up and distributed to everybody around the house”* (RES01). Other occasions such as the Queen’s birthday, Easter and national sporting events were also frequently marked with a special meal and event-specific dining room decorations. The celebration of special occasions was appreciated by participants:

*“I mean, they're very good here, because the other day we had a Wimbledon lunch, which was lunch in a basket, followed by strawberries and cream, a scone, and a Pimms No.1.”* (RES08)

There was a sense that special meals or celebratory occasions offer a welcome break from routine, as well as providing an opportunity for conversation and shared ritual. Indeed, the concept of routine required that a balance be struck between the comfort gleaned from having the same breakfast every morning or sitting in the same seat, and the feelings of institutionalisation associated with perpetual routine. This theme also highlighted how special meals could evoke memories of the past, and in so doing induce a sense of familiarity. For instance, residents spoke of how the opportunity to eat fish and chips in newspaper reminded them of good times, made them feel at home, and nurtured a closeness among residents.

## Discussion

This study has highlighted the importance of social interaction between residents, as well as the importance of accommodating personal preferences, which are often shaped by the habitual and traditional dimensions of mealtimes. Catering for individual preferences can be problematic when faced with the competing interests of residents and the limitations of collective care provision. The findings demonstrate the complexity of mealtimes as experienced by care home residents, and have revealed some of the ‘active ingredients’ that may contribute to effective mealtime interventions [35].

The transition from independent living to life in a care home can be a stressful experience for new residents, who may feel helpless and abandoned [36], and be confused, anxious and depressed [37]. This may result from a sense of discontinuity between former and present lives, and the lack of privacy and autonomy may lead to social isolation and loneliness [38]. Autonomy and self-efficacy are also undermined by having key decisions made routinely for residents [39]. Entrusting residents to make decisions about aspects of their care may help to ease the transition by reducing feelings of disempowerment and boosting self-efficacy. The current findings support the notion that individual choice and freedom to express personal preferences are key components of residents’ experiences of care; mealtimes are a focal point of these experiences and provide a social context through which individual needs can be realised. Consistent with this view, research by Haslam et al. (2012) has shown how giving residents greater control over the decision-making process strengthens their sense of community, or shared social identity with others which, in turn, promotes social interaction, greater engagement and wellbeing [40], and also a wider sense of citizenship [41].

A Care Quality Commission (CQC) inspection programme, which audited 500 care homes in the UK in 2012, found that one in six care homes (80 homes) did not always respect the privacy and dignity of residents or involve them in their care, failing for example to provide a choice of activities and options for residents or support their independence [3]. Such findings are at clear odds with the resident preferences as expressed in the current study. Mealtimes in particular are an opportunity for residents to exercise some control over part of their life in care, for example through deciding what to eat, where to eat, when to eat and with whom [42]. This is acknowledged by the British Geriatrics Society (2011), which highlights the importance of involving residents in decisions about their care, including aspects of care relating to mealtimes [4]. However, despite evidence that such control is positively associated with quality of life [16], it is often at odds with the routinized, communal organisation of many institutions [16, 43, 44].

Given the challenge of accommodating individual needs and preferences in a communal context, our findings highlight the importance of striking the right balance at mealtimes. On the one hand, our study shows that there is a balance to be struck between routine and novelty. For example, whilst participants described being “creatures of habit” at mealtimes, they also appreciated the break from routine effectuated by the marking of special occasions. On the other hand, there was a perennial juxtaposition between individual and group interests, as exemplified by meal options or by seating allocation. In the wider literature, mealtimes have been described as offering a sense of social normality with residents sharing food, passing condiments and pouring drinks for each other, thereby contributing to a feeling of belonging [16, 42] and possibly enhancing a sense of community. At the same time, some residents clearly prefer privacy (i.e., eating alone) at mealtimes with ‘homeliness’ associated more closely with family than with fellow residents of staff [45]. Searching for balance in this scenario might involve altering the dining room to create a more intimate atmosphere, or offering residents the opportunity to eat in their own room. Although eating alone is inconsistent with the notion of group or social dining [46], safeguarding residents’ privacy may enable them to identify with the collective if being private in normatively approved [47] – satisfying individual and group interests.

The notion of balance is closely related to choice: How much choice can residents be afforded at mealtimes? Our participants described having little or no choice over where to sit in the dining room and meal-times were generally fixed. This is in contrast to normal eating behaviour (and mealtimes) which are defined by choice (or negotiated choices). Indeed, food enhances socialisation (and wellbeing) in the real world because people choose what to eat, when to eat and who to eat with [48]. Despite this, there is scant research on tablemate interactions, flexible mealtimes, and on other preferences such as where to eat [20]. As much of the care given to older adults in care homes is prescribed, opportunities to defer choice to residents, such as what, where and when to eat, may help to improve the dining experience.

### Limitations

Care homes were sampled on the basis that they were rated as providing a good overall standard of care, and managers may have decided to participate in the study because they had a high level of confidence in their provision of care. The experiences of residents in less well performing care homes may be different. For example, it is possible that poorly-rated care homes might provide poorer quality meals or inadequate dining facilities. Resident experiences in such scenarios may be more likely reflect these deficiencies. Moreover, interviews were conducted with eleven willing participants. While there was considerable commonality in current residents’ responses, other residents in these care homes might have expressed different views. Finally, all of the participant care homes were located in the South West of England, which may not reflect the cultural and ethnic diversity characteristic of other areas of the UK and beyond.

Qualitative studies may incorporate member checking or use an external auditor to improve the quality of the research result. However, due to time and resource constraints, it was not possible to use these methods in this study.

### Implications for future research

The Medical Research Council (MRC) emphasises the importance of developing a cumulative understanding of how complex interventions work so that their effectiveness can be enhanced and applied across a diverse range of groups and settings [14]. Previous research into mealtime interventions in care homes has tended to focus on single-component interventions and has lacked the rigour and validity merited by the complexity of the population and setting [12, 13, 49]. The current study has highlighted the complexity of the mealtime experience and the need for interventions to account for this. In particular, our findings suggest that future studies should focus on resident choice at mealtimes. For instance, there may be benefits to involving residents, meaningfully and collectively, in decisions about mealtimes, from meal planning and preparation, to seating arrangements. Collective decisions about the social environment may be a simple way of making residents feel “at home”, thereby enhancing their psychological functioning [40]. Collective engagement, specifically the involvement of residents in group activities, has been posited as a means of building social relations within the home, alleviating residents’ sense of confinement and gaining back some control. Mealtimes that promote a social environment and a convivial atmosphere may improve mood and appetite, add meaning and structure to the day, and contribute to a greater sense of satisfaction with life [50, 51]. In addition, we found that mealtime routines were valuable, but occasional variety was necessary. On the basis of participant responses, it seems important to identify and evaluate ways to introduce variety during mealtimes. For instance, should variety be incorporated by celebrating shared holidays or by allowing choice?

## Conclusion

This research highlights the importance of understanding residents’ routines, habits and preferences from the point at which they begin life in the care home, ensuring they are empowered to make their own decisions where possible and providing a dining environment that is social, convivial, and enjoyable. Residents’ experiences of mealtimes may provide important insight into these psychosocial influences on health and wellbeing and future interventions could consider how the physical health outcomes of residents are impacted by the social and psychological components highlighted in this study.

## References

[1] Care Quality Commission. The Adult Social Care Market and the Quality of Services. 2010.

[2] Age UK. Later Life in the United Kingdom 2015. Available from: http://www.ageuk.org.uk/Documents/EN-GB/Factsheets/Later_Life_UK_factsheet.pdf?dtrk=true.

[3] Care Quality Commission. Time to listen in care homes 2012 [cited 2015 November 11th]. Available from: https://www.cqc.org.uk/sites/default/files/documents/time_to_listen_-_care_homes_main_report_tag.pdf.

[4] British Geriatrics Society. Quest for Quality. British Geriatrics Society, 2011. Available from: http://www.bgs.org.uk/campaigns/carehomes/quest_quality_care_homes.pdf.

[5] Macleod J, Smith GD (2003). Psychosocial factors and public health: a suitable case for treatment?. J Epidemiol Community Health.

[6] Patrick DL, Kinne S, Engelberg RA, Pearlman RA (2000). Functional status and perceived quality of life in adults with and without chronic conditions. J Clin Epidemiol.

[7] McDougall FA, Matthews FE, Kvaal K, Dewey ME, Brayne C (2007). Prevalence and symptomatology of depression in older people living in institutions in England and Wales. Age Ageing.

[8] Donini LM, Savina C, Cannella C (2003). Eating habits and appetite control in the elderly: the anorexia of aging. Int Psychogeriatr.

[9] Volicer L, Frijters DH, van der Steen JT (2013). Apathy and weight loss in nursing home residents: longitudinal study. J Am Med Dir Assoc.

[10] Savishinsky J. Bread and butter” issues: Food, conflict, and control in a nursing home. Gray Areas: Ethnographic Encounters with Nursing Home Culture. New Mexico: School of American Research Press; 2003:103–20.

[11] Watkins R, Goodwin VA, Abbott RA, Backhouse A, Moore D, Tarrant M. Attitudes, perceptions and experiences of mealtimes among residents and staff in care homes for older adults: A systematic review of the qualitative literature. Geriatr Nurs. 2017. 10.1016/j.gerinurse.2016.12.002 28089317

[12] Abbott R, Whear R, Thompson-Coon J, Ukoumunne OC, Bethel A, Rogers M, Hemsley A, Stein K (2013). Effectiveness of mealtime interventions on nutritional outcomes for the elderly living in residential care: A systematic review and meta-analysis. Ageing Res Rev.

[13] Whear R, Abbott R, Thompson-Coon J, Bethel A, Rogers M, Hemsley A, Stahl-Timmins W, Stein K (2014). Effectiveness of Mealtime Interventions on Behavior Symptoms of People With Dementia Living in Care Homes: A Systematic Review. JAMDA.

[14] Craig P, Dieppe P, Macintyre S, Michie S, Nazareth I, Petticrew M (2008). Developing and evaluating complex interventions: the new Medical Research Council guidance. BMJ.

[15] Grodner M, Anderson S, De Young S (2000). Foundations and clinical applications of nutrition: A nursing approach.

[16] Philpin S, Merrell J, Warring J, Hobby D, Gregory V (2014). Memories, identity and homeliness: The social construction of mealtimes in residential care homes in South Wales. Ageing Soc.

[17] Evans B, Crogan N, Shultz J (2005). The meaning of mealtimes: Connection to the social world on the nursing home. J Gerontol Nurs.

[18] Vucea V, Keller HH, Ducak K (2014). Interventions for improving mealtime experiences in long-term care. J Nutr Gerontol Geriatr.

[19] Bradshaw SA, Playford ED, Riazi A (2012). Living well in care homes: A systematic review of qualitative studies. Age Ageing.

[20] Reimer HD, Keller HH (2009). Mealtimes in Nursing Homes: Striving for Person-Centred Care. J Nutr Elder.

[21] Commission CQ. The State of Health Care and Adult Social Care in England: Key themes and quality of services in 2009. The Stationery Office. 2010.

[22] Pearson A, FitzGerald M, Nay R (2003). Mealtimes in nursing homes: the role of nursing staff. J Gerontol Nurs.

[23] Sidenvall B (1999). Meal procedures in institutions for elderly people: a theoretical interpretation. J Adv Nurs.

[24] Mays N, Pope C (1996). Rigour in qualitative research.

[25] Barbour RS (2001). Checklists for improving rigour in qualitative research: a case of the tail wagging the dog?. Br Med J.

[26] Guest G, Bunce A, Johnson L (2006). How Many Interviews Are Enough? An Experiment with Data Saturation and Variability. Field Methods.

[27] Pope C, Mays N (1995). Reaching the parts other methods cannot reach: an introduction to qualitative methods in health and health services research. Br Med J.

[28] Patton MQ (1990). QualitW and research methods.

[29] Glaser BG, Strauss AL (1967). The discovery of grounded theory: strategies for qualitative research.

[30] Geertz C (1973). Thick description: Toward an Interpretative Theory of Culture.

[31] Braun V, Clarke V (2006). Using thematic analysis in psychology. Qual Res Psychol.

[32] Attride-Stirling J (2001). Thematic networks: an anaytic tool for qualitative research. Qual Res.

[33] Koch T, Harrington A (1998). Reconceptualizing rigour: the case for reflexivity. J Adv Nurs.

[34] Mays N, Pope C (2000). Assessing quality in qualitative research. Br Med J.

[35] Mitchie S, Abraham C (2004). Interventions to change health behaviours: evidence-based or evidence-inspired?. Psychol Health.

[36] Kao HFS, Travis SS, Acton GJ (2004). Relocation to a long-term care facility: Working with patients and families before, during, and after. J Psychosoc Nurs Ment Health Serv.

[37] Hodgson N, Freedman VA, Granger DA, Erno A (2004). Biobehavioral correlates of relocation in the frail elderly: salivary cortisol, affect, and cognitive function. J Am Geriatr Soc.

[38] Choi NG, Ransom S, Wyllie RJ (2008). Depression in older nursing home residents: The influence of nursing home environmental stressors, coping, and acceptance of group and individual therapy. Aging Mental Health.

[39] Hauge S, Heggen K (2007). The nursing home as a home: a field study of residents’ daily life in the common living rooms. J Clin Nurs.

[40] Haslam C, Jetten J, Haslam SA, Knight C (2012). The importance of remembering and deciding together: Enhancing the health and well-being of older adults in care.

[41] Knight C, Haslam SA, Haslam C (2010). In home or at home? How collective decision making in a new care facility enhances social interaction and wellbeing amongst older adults. Ageing Soc.

[42] Palacios-Cena D, Losa-Iglesias ME, Cachon-Perez JM, Gomez-Perez D, Gomez-Calero C, Fernandez-de-las-Penas C (2012). Is the mealtime experience in nursing homes understood? A qualitative study. Geriatr Gerontol Int.

[43] Dunn H, Moore T. ‘You can’t be forcing food down ‘em’: Nursing home carers’ perceptions of residents’ dining needs. J Health Psychol. 2014; doi:10.1177/1359105314532971. 10.1177/1359105314532971 24829377

[44] Harnett T, Jönson H. Shaping nursing home mealtimes. Ageing Soc. 2015:1–22.

[45] Bennett MK, Ward EC, Scarinci NA (2015). Mealtime management in Australian residential aged care: Comparison of documented, reported and observed care. Int J Speech Lang Pathol.

[46] Amella EJ (2004). Feeding and hydration issues for older adults with dementia. Nurs Clin N Am.

[47] Jetten J, Haslam C, Alexander SH. The social cure: Identity, health and well-being. Psychology Press. 2012.

[48] Crogan NL, Evans B, Severtsen B, Shultz JA (2004). Improving nursing home food service: uncovering the meaning of food through residents’ stories. J Gerontol Nurs.

[49] Keller H, Carrier N, Duizer L, Lengyel C, Slaughter S, Steele C (2014). Making the Most of Mealtimes (M3): grounding mealtime interventions with a conceptual model. JAMDA.

[50] Njis K, de Graaf C, Kok FJ, Van Staveren WA (2006). Effect of family-style mealtimes on quality of life, physical performance, and body weight of nursing home residents: cluster randomised controlled trial. Br Med J.

[51] Njis K, de Graaf C, Siebelink E, Blauw YH, Vanneste V, Kok FJ, Van Staveren WA (2006). Effect of family-style meals on energy intake and risk of malnutrition in Dutch nursing home residents: a randomized controlled trial. J Gerontol.

